# Single‐molecule real‐time sequencing reveals diverse allelic variations in carotenoid biosynthetic genes in pepper (*Capsicum* spp.)

**DOI:** 10.1111/pbi.13039

**Published:** 2018-12-09

**Authors:** Hyo‐Bong Jeong, Min‐Young Kang, Ayoung Jung, Koeun Han, Joung‐Ho Lee, Jinkwan Jo, Hea‐Young Lee, Jong‐Wook An, Suna Kim, Byoung‐Cheorl Kang

**Affiliations:** ^1^ Department of Plant Science Plant Genomics & Breeding Institute Research Institute of Agriculture and Life Sciences Seoul National University Seoul Korea; ^2^ Food and Nutrition in Home Economics Korea National Open University Seoul Korea; ^3^ Crop Biotechnology Institute/GreenBio Science and Technology Seoul National University Seoul Korea

**Keywords:** *Capsicum*, carotenoid, SMRT sequencing, phytoene synthase, capsanthin‐capsorubin synthase

## Abstract

The diverse colours of mature pepper (*Capsicum* spp.) fruit result from the accumulation of different carotenoids. The carotenoid biosynthetic pathway has been well elucidated in Solanaceous plants, and analysis of candidate genes involved in this process has revealed variations in carotenoid biosynthetic genes in *Capsicum* spp. However, the allelic variations revealed by previous studies could not fully explain the variation in fruit colour in *Capsicum* spp. due to technical difficulties in detecting allelic variation in multiple candidate genes in numerous samples. In this study, we uncovered allelic variations in six carotenoid biosynthetic genes, including phytoene synthase (*PSY1*, *PSY2*), lycopene β‐cyclase, β‐carotene hydroxylase, zeaxanthin epoxidase and capsanthin‐capsorubin synthase (*CCS*) genes, in 94 pepper accessions by single‐molecule real‐time (SMRT) sequencing. To investigate the relationship between allelic variations in the candidate genes and differences in fruit colour, we performed ultra‐performance liquid chromatography analysis using 43 accessions representing each allelic variation. Different combinations of dysfunctional mutations in *PSY1* and *CCS* could explain variation in the compositions and levels of carotenoids in the accessions examined in this study. Our results demonstrate that SMRT sequencing technology can be used to rapidly identify allelic variation in target genes in various germplasms. The newly identified allelic variants will be useful for pepper breeding and for further analysis of carotenoid biosynthesis pathways.

## Introduction

The diverse colours of mature pepper fruit result from the accumulation of carotenoids in parallel with the degradation of chlorophyll. Carotenoids are a subgroup of isoprenoids that typically contain 40 carbons in their polyene backbones (Delgado‐Vargas *et al*., 2000; Rosati *et al*., 2009). Carotenoids play diverse roles in essential processes in plants, including photosynthesis and photo‐protection (Domonkos *et al*., 2013). These compounds also serve as precursors for the biosynthesis of phytohormones such as abscisic acid (ABA) and strigolactones (Al‐Babili and Bouwmeester, 2015; Nambara and Marion‐Poll, 2005). The carotenoid biosynthetic pathway and the associated enzymes are well elucidated in Solanaceous plants. Carotenoid biosynthesis begins with the formation of phytoene by phytoene synthase (PSY). Following a series of desaturation and isomerization steps, phytoene is converted to lycopene, the major carotenoid component in mature tomatoes (Cunningham and Gantt, 1998). While the red colour of tomato fruit is due to the accumulation of lycopene, the red colour in pepper fruit results from the accumulation of capsanthin and capsorubin (Paran and van der Knaap, 2007). This *Capsicum*‐specific process is controlled by capsanthin‐capsorubin synthase (CCS), which converts antheraxanthin and violaxanthin into capsanthin and capsorubin respectively (Bouvier *et al*., 1994).

In pepper, a three‐locus model (*Y*, *C1* and *C2*) was originally proposed for the inheritance of fruit colour (Hurtado‐Hernandez and Smith, 1985). Analyses of candidate carotenoid biosynthetic genes in various fruit colour loci revealed that the *Y* and *C2* loci harbour *CCS* and *PSY1* respectively (Huh *et al*., 2001; Lefebvre *et al*., 1998; Popovsky and Paran, 2000) but the third locus *C1* remains to be discovered. Since the elucidation of these loci, many researchers have tried to explain the fruit colour variation in pepper based on allelic variation in *PSY1* and *CCS*. In general, peppers with yellow fruit have structural mutations in the coding region of *CCS* that lead to a premature stop codon (Ha *et al*., 2007; Lefebvre *et al*., 1998; Li *et al*., 2013; Popovsky and Paran, 2000). However, early translational termination of *CCS* can also result in orange fruit, as in *C. annuum ‘*Fogo’ (Guzman *et al*., 2010). These findings imply that allelic variation in *CCS* does not lead to a specific colour, although mutations in *CCS* significantly affect fruit colour. In contrast, Kim *et al*. (2010) showed that a splicing mutation in *PSY1* impairs the activity and leads to the production of orange fruits in *C. chinense* ‘Habanero’. Taken together, the allelic variations in *PSY1* and *CCS* revealed in previous studies do not fully explain the fruit colour variation in *Capsicum* spp. To find the last colour‐determining locus and complement the three‐locus model, many studies have investigated the roles of other carotenoid biosynthetic genes in determining fruit colour. Virus‐induced gene silencing (VIGS) of capsanthin biosynthetic genes in detached red pepper fruit revealed that not only *PSY1* and *CCS* but also *Lcyb* and *CrtZ‐2* are associated with carotenoid biosynthesis (Tian *et al*., 2014). In addition, Borovsky *et al*. (2013) found that an EMS‐induced nucleotide substitution in the coding region of *CrtZ‐2* led to the conversion of red fruit to orange fruit in *C. annuum* ‘Maor’. Moreover, in a tomato mutant with increased pigmentation, designated as a *high‐pigment 3*, amino acid substitution in the conserved domain of *ZEP* was uncovered (Galpaz *et al*., 2008). Therefore, allelic variation in candidate carotenoid biosynthetic genes should be thoroughly investigated to better understand fruit colour variation in *Capsicum* spp.

Next‐generation sequencing (NGS) technologies have enabled the characterization of entire genomes and have revolutionized genetic studies and crop breeding (Varshney *et al*., 2009). NGS technologies can be also used to study genetic variation in candidate genes or specific genomic regions in numerous samples (Guo *et al*., 2015). Targeted sequencing can be accomplished by performing primary amplification with target specific primers and tagging individual samples with barcode sequences in the next round of amplification (Yang *et al*., 2015). However, the short read lengths obtained by Illumina‐based NGS prevent the efficient acquisition of full‐length target sequences from many samples. This problem can be addressed using single‐molecule real‐time (SMRT) sequencing developed by Pacific BioSciences (Menlo Park, CA). SMRT sequencing provides much longer read lengths (over 10 kb) and faster run times than any other NGS technology. If the length of a target gene is less than 10 kb, target sequences can be obtained in a single SMRT sequencing run (Rhoads and Au, 2015).

In this study, we rapidly analysed the coding regions of *PSY1*, *PSY2*, *Lcyb*, *CrtZ‐2*, *ZEP* and *CCS* in 94 pepper accessions using SMRT sequencing and detected diverse allelic variation in these genes. Most of the alleles identified in this study are novel alleles that have not been reported previously. In addition, we identified new structural mutations in the *CCS* promoter region of non‐red pepper accessions by PCR analysis. We performed ultra‐performance liquid chromatography (UPLC) of carotenoids from representative accessions to investigate the relationship between allelic variations in the candidate genes and fruit colour. Different combinations of dysfunctional mutations of *PSY1* and *CCS* could explain the variation in carotenoid contents in non‐red fruits. Noticeably, in specific chromatogram group possessing *PSY1/ccs* genotype, two subgroups could be obtained according to the levels of carotene, cryptoxanthin and zeaxanthin. It implies the presence of a gene with unknown function, perhaps, *C1* locus. Our findings demonstrate that SMRT sequencing can be successfully used to identify genetic variation in target regions, even in a large number of samples. The allelic variation identified in this study provides useful information about carotenoid biosynthesis and could facilitate pepper breeding.

## Results

### Library construction for SMRT sequencing

**Figure 1:**
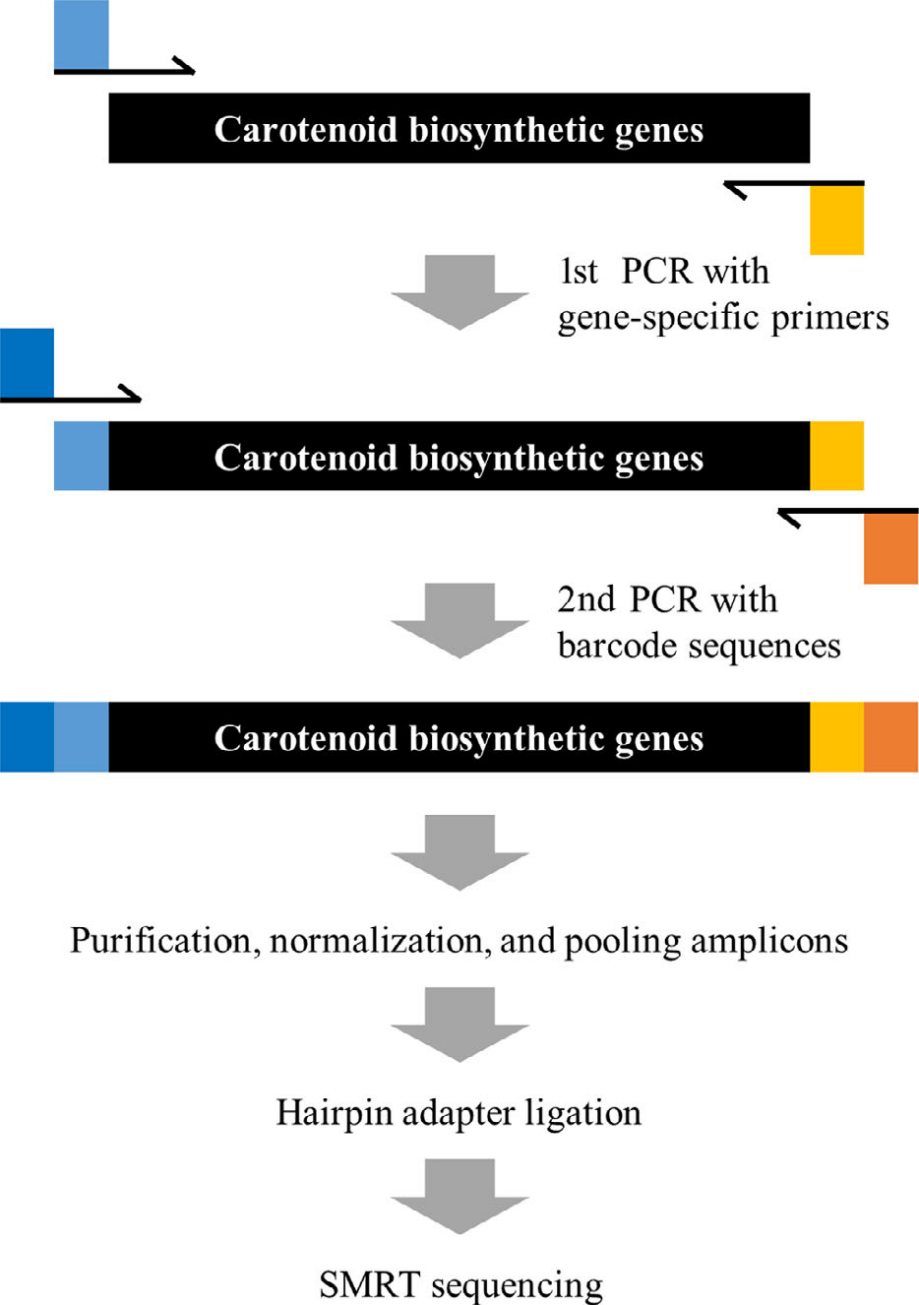
Schematic diagram of library construction for single‐molecule real‐time (SMRT) sequencing. The SMRT sequencing process consists of five steps. The target gene was amplified using gene‐specific primers with universal tags. The amplicon template was re‐amplified using anti‐tag universal primers with sample‐specific barcode sequences. The library was constructed by pooling the amplicons. After ligating hairpin adapters to allow continuous circular sequencing, templates were subjected to be sequenced. Light blue and light orange squares represent universal tags used in the first PCR, and deep blue and deep red squares represent barcode sequences used in the second PCR.

We constructed SMRT sequencing libraries by amplifying six carotenoid biosynthesis genes, including *PSY1, PSY2, Lcyb, CrtZ‐2, ZEP* and *CCS* (Figure 1 and Table 1)*. PSY1* consists of six exons with a 1260 bp coding sequence. Two sets of primers corresponding to the N‐ and C‐terminal regions (1695 and 1690 bp) of *PSY1* were designed for the first round of PCR. Among the 94 accessions, we obtained amplicons of the expected size for 75 and 74 accessions in the N‐ and C‐terminal region of *PSY1*. We obtained an amplicon of an unexpected size for one *C. baccatum* accession (ACN 64) in both termini, and no amplicons were obtained for 18 and 19 of the remaining accessions for the N‐ and C‐terminal regions respectively. *PSY2* consists of six exons with a 1299 bp coding sequence. Two sets of primers targeting the N‐ and C‐terminal regions (1623 bp in both cases) of *PSY2* were designed for the primary PCR. Every accession examined showed expected pattern of amplification with the predicted size of bands. *Lcyb* has a single exon 1497 bp in size. We designed primers for *Lcyb* to amplify a 1706 bp fragment. Amplicons of the expected size were obtained for 90 accessions, whereas no amplicon was obtained for four *C. annuum* accessions (ACN 4, 15, 53 and 58). *CrtZ‐2* is composed of seven exons with a coding sequence 948 bp in size. Two sets of primers corresponding to the N‐ and C‐terminal region (1677 and 1634 bp) were developed for the amplification of *CrtZ‐2*. For the N‐terminal region, amplicons of the expected size were obtained for 75 samples, whereas larger than expected amplicons were obtained for 17 accessions and smaller amplicons were obtained for one *C. annuum* (ACN 27) and one *C. baccatum* (ACN 69) accession. All samples produced amplicons of the expected size for the C‐terminal region of this gene. *ZEP* consists of 16 exons with a coding sequence 1986 bp in size. For amplification of *ZEP*, three sets of primers corresponding to both termini along with central region (1760, 1709 and 1704 bp) were developed. All accessions were normally amplified in all three region of *ZEP* without exception. Finally, *CCS* has a single exon 1497 bp in size. Primers for *CCS* were designed to amplify a 1674 bp fragment. Amplicons of the expected size were obtained for 62 samples, whereas no amplicon was obtained for 32 samples (Table S3). We performed the secondary PCR using amplicons of the expected size with primers containing barcode sequences for individual samples and pooled the amplicons for SMRT sequencing (Figure 1).

**Table 1:** Information about the target genes and the number of mutations detected in non‐red pepper fruit by single‐molecule real‐time sequencing

Gene	Gene no.	CDS (bp)	gDNA (bp)	Number of mutations		
				Mis‐sense	Non‐sense	Indel
*PSY1*	CA04g04080	1260	2844	9	1	2
*PSY2*	CA02g20350	1299	2985	3	0	0
*Lcyb*	CA05g00080	1497	1497	6	0	1
*CrtZ‐2*	CA03g25820	948	2025	9	0	0
*ZEP*	CA02g10990	1986	4802	16	0	0
*CCS*	CA06g22860	1497	1497	3	4	6

### SMRT sequencing and allelic variation

Single‐molecule real‐time sequencing was performed using two SMRT cells in order to obtain sufficient depth of sequencing data in all six carotenoid biosynthetic genes. In the first cell corresponding to *PSY1*, *Lcyb*, *CrtZ‐2* and *CCS*, SMRT sequencing yielded 300 584 raw reads, which were narrowed down to 94 318 reads after filtering. We obtained 1 278 193 633 bp of sequence with a mean read length of 13 551 bp. In the second cell targeting *PSY2* and *ZEP*, 150 292 raw reads were obtained. Among them, 108 181 reads were left after filtering. A total of 2 041 520 976 bp were confirmed with 18 871 bp of average read length (Table S4). We aligned sub‐reads to regions in *PSY1*, *PSY2*, *Lcyb*, *CrtZ‐2, ZEP* and *CCS* and retained variants with minimum coverage of 20 and minimum confidence of 20 for further analysis. SNP and indel mutations found in red fruits were excluded from further analysis.

#### Allelic variation in *PSY1*

**Figure 2:**
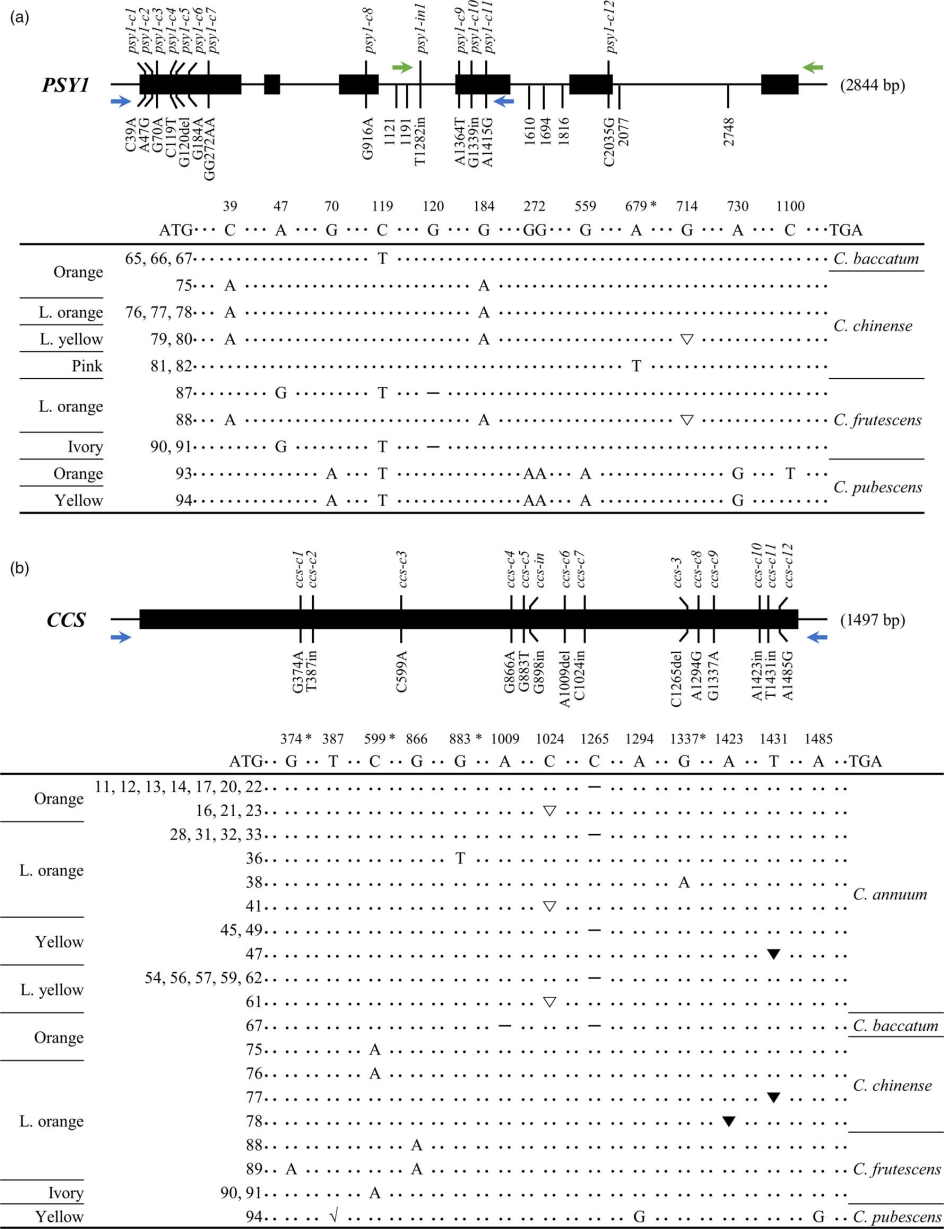
*PSY1*/*CCS* gene structure and comparisons of the coding regions in various *Capsicum* accessions. Black rectangles represent exons and solid lines represent introns. Mutations in exon regions that can change the amino acid sequence are described along with allele names. Indel mutations occurred in intron regions are only indicated by their locations. In schematic representation of mutations in the coding region, ATG and TGA represent the start and stop codons, respectively, and the numbers indicate the nucleotide positions in cDNA. (a) *PSY1* consists of six exons and five introns. Thirteen mutant alleles were detected in accessions with non‐red fruit. Asterisk indicates the formation of a stop codon by non‐sense mutations. Hyphen (–) indicates a 1 bp deletion in the coding sequence, which leads to a premature stop codon. The 2 bp insertion in the coding sequence, which leads to a frame‐shift, is indicated by an empty triangle (∇). (b) *CCS* contains a single exon. Thirteen mutant alleles were detected in non‐red fruit accessions. Asterisk indicates the formation of a stop codon by non‐sense mutations. Hyphen (–) indicates a 1 bp deletion in the coding sequence, which leads to an early translational termination. ∇, ▼ and √ represent a 1, 8 and 76 bp insertion in the nucleotide sequence, respectively, which lead to a frame‐shift. *CCS*, capsanthin‐capsorubin synthase.

Single‐molecule real‐time sequencing revealed 36 and 60 different mutations in the exons and introns of *PSY1* respectively (Table S5). Among these 96 variations, we identified mutations in exon regions that result in changes in amino acid sequences and indel mutations in intron regions, as shown in Figure 2a. The allelic variations in exon regions were named according to the position of the mutated nucleotide in the coding sequence. Among the 12 mutations located in the coding sequence of *PSY1*, nine were mis‐sense mutations. The three remaining allelic variations including two frame‐shift mutations at the first and fourth exons and one non‐sense mutation at the fourth exon resulted in the knockout of *PSY1*. A frame‐shift mutation due to a 1 bp deletion at the 120 nucleotide (nt) position, designated *psy1‐c5*, was detected in three *C. frutescens* accessions (ACN 87, 90 and 91), and the other frame‐shift mutation (*psy1‐c10*), which was due to a 2 bp insertion at position 714, was detected in two *C. chinense* accessions (ACN 79 and 80) and one *C. frutescens* accession (ACN 88). Finally, *psy1‐c9*, a non‐sense mutation at position 679, was only detected in two *C. chinense* accessions (ACN 81 and 82).

#### Allelic variation in *PSY2*

**Figure 3:**
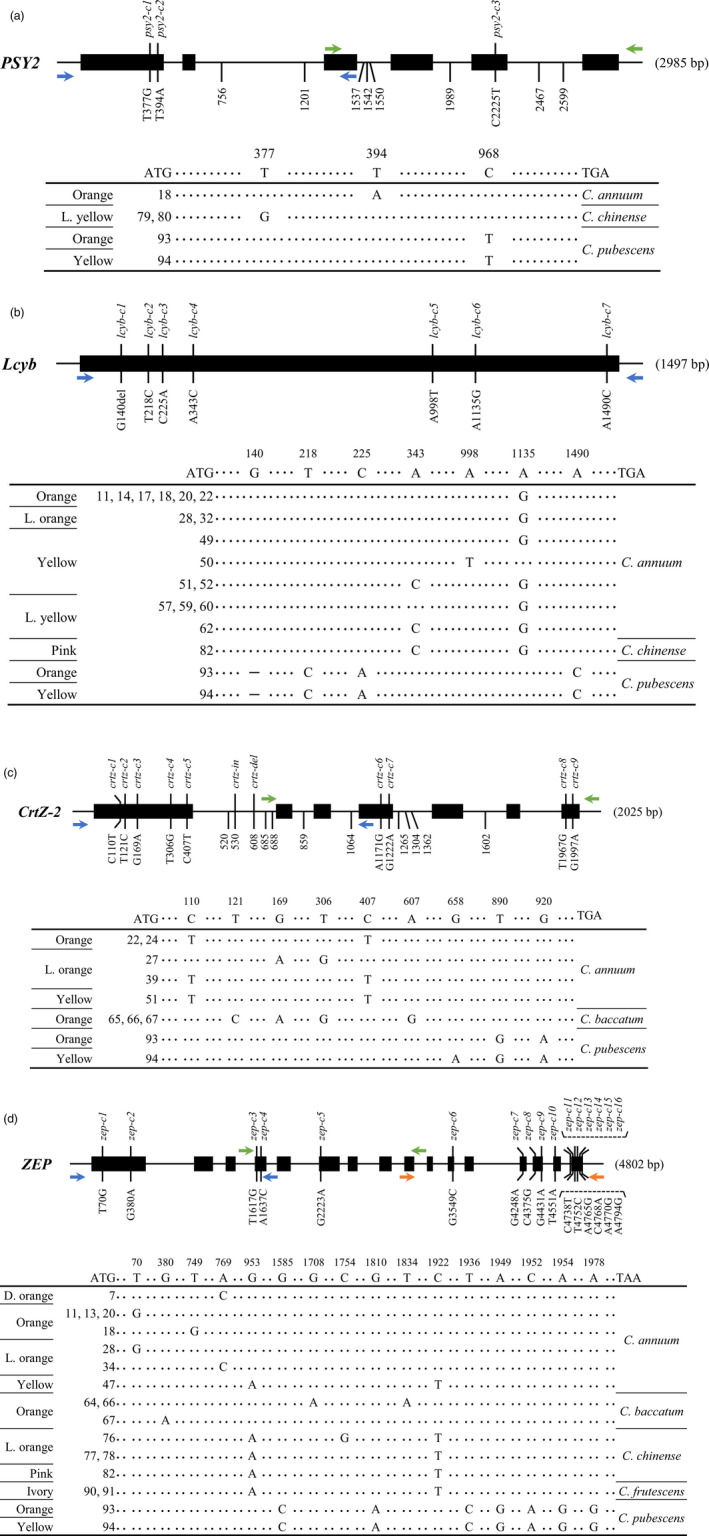
*PSY2*/*Lcyb*/*CrtZ‐2*/*ZEP* gene structure and comparisons of the coding regions in various *Capsicum* accessions. Black rectangles depict exons and solid lines depict introns. Mutations in exon regions which are predicted to affect the amino acid changes are described along with allele names. Indel mutations in introns are only represented by their locations (except for *ZEP* due to the lack of sufficient space in diagram). In schematic representation of mutations in the coding region, ATG and TGA/TAA represent the start and stop codons, respectively, and the numbers indicate the nucleotide positions in cDNA. (a) *PSY2* consists of six exons and five introns. Three mutant alleles were found in accessions with non‐red fruit. (b) *Lcyb* contains a single exon and seven allelic variations were detected in non‐red fruit accessions. Hyphen (–) indicates a 12 bp deletion in the coding region. (c) *CrtZ‐2* is composed of seven exons and six introns. A total of eleven variations were uncovered. (d) *ZEP* consists of 16 exons and 15 introns. In total, 16 mutant alleles were found [Correction added on 8 January 2019, after first online publication. Information regarding Figures 3a,b,d, and Figures 4 and 5 were previously incorrect and are now updated in this version.].

In total, 7 and 20 mutations were uncovered in the exon and intron regions of *PSY2* by SMRT sequencing (Table S5). Remarkably, the number of investigated mutations were considerably low compared to other genes. Of seven mutations in coding sequences, only the three mutations were predicted to change the amino acid sequence of *PSY2* without causing premature stop codons (Figure 3a). A mis‐sense mutation occurred at the first exon (*psy2‐c1*) was detected in two *C. chinense* accessions (ACN 79 and 80) and another mis‐sense mutation at the fifth exon, designated as *psy2‐c3*, was found in two *C. pubescens* accessions (ACN 93 and 94).

#### Allelic variation in *Lcyb*

Single‐molecule real‐time sequencing revealed 31 different allelic variations in *Lcyb* in the 94 accessions (Table S5), only seven of which resulted in changes in amino acid sequences (Figure 3b). Most of these mutations were mis‐sense mutations. However, *lcyb‐c1*, a frame‐shift mutation due to a 12 bp deletion at 140 nt, was detected in two *C. pubescens* accessions (ACN 93 and 94). Moreover, a *C. annuum*‐specific allele (*lcyb‐c6*) with an amino acid substitution from asparagine to aspartic acid at amino acid 379 position was detected across 16 accessions. Accessions possessing this mutation have fruits with a wide range of colours, from pink to orange.

#### Allelic variation in *CrtZ‐2*

A total of 16 and 58 mutations were detected in the exons and introns of *CrtZ‐2* respectively (Table S5). Of the 16 mutations in coding sequences, nine mutations were expected to lead to changes in amino acid sequence and none were non‐sense mutations (Figure 3c). Two mis‐sense mutations, *crtz‐c1* and *crtz‐c5*, were highly conserved in four *C. annuum* accessions (ACN 22, 24, 39 and 51). Four mis‐sense mutations, *crtz‐c2*, *crtz‐c3*, *crtz‐c4* and *crtz‐c6*, were detected in three *C. baccatum* accessions (ACN 65, 66 and 67), and two other mis‐sense mutations, *crtz‐c8* and *crtz‐c9*, were detected in two *C. pubescens* accessions (ACN 93 and 94).

#### Allelic variation in *ZEP*

In total, 30 and 61 mutations were detected in the exon and intron regions of *ZEP* by SMRT sequencing (Table S5). Among 30 mutations in coding sequences, 16 mutations leads to the amino acid changes. Such variations all belong to mis‐sense mutations (Figure 3d). Seven mutations, including *zep‐c6*, *zep‐c9*, *zep‐c12*, *zep‐c13*, *zep‐c14*, *zep‐c15* and *zep‐c16*, were exclusively detected in two *C. pubescens* accessions (ACN 93 and 94). Whereas, another two mutations (*zep‐c5* and *zep‐c11*) were revealed in diverse species including one *C. annuum* (ACN 47), four *C. chinense* (ACN 76, 77, 78 and 82) and two *C. frutescens* (ACN 90 and 91) accessions.

#### Allelic variation in *CCS*

Single‐molecule real‐time sequencing revealed 29 mutations in *CCS* (Table S5). Among these, 13 variations including six frame‐shift mutations, four non‐sense mutations and three mis‐sense mutations resulted in amino acid changes (Figure 2b). The most frequently detected frame‐shift mutation was *ccs‐3*, which was previously named by Guzman *et al*. (2010). This mutation, which was caused by a 1 bp deletion at position 1265 nt, was found in 18 *C. annuum* accessions (Table S6) and one *C. baccatum* accession (ACN 67). The next most common frame‐shift mutation (*ccs‐c7*) occurred at position of 1024 nt due to a 1 bp insertion, which was found only in *C. annuum* accessions (ACN 16, 21, 23, 41 and 61). Another frame‐shift mutation due to a 8 bp insertion at 1431 nt (*ccs‐c11*) was detected in one *C. annuum* (ACN 47) and one *C. chinense* accession (ACN 77). Among non‐sense mutations, *ccs‐c3* at position 599 nt was found in two *C. chinense* (ACN 75 and 76) and two *C. frutescens* accessions (ACN 90 and 91). However, it was difficult to identify a species‐specific trend for the remaining mutations, as the number of accessions containing these mutations was limited. Three frame‐shift mutations, including *ccs‐c2*, *ccs‐c6* and *ccs‐c10*, were found only in a single accession (ACN 94, 67 and 78 respectively). Likewise, three non‐sense mutations, *ccs‐c1*, *ccs‐c5* and *ccs‐c9*, were only detected in a single accession (ACN 89, 36 and 38 respectively). Among the 10 knockout mutations, *ccs‐c3*, *ccs‐3* and *ccs‐c11* were previously reported: *ccs‐c3* was identified in *C. chinense* ‘IT164918’ (Ha *et al*., 2007), *ccs‐3* was found in *C. annuum* ‘Fogo’ and ‘CK7’ (Guzman *et al*., 2010; Li *et al*., 2013) and *ccs‐c11* was detected in *C. chinense* ‘IT800065’ (Ha *et al*., 2007). These findings are consistent with the current results, as these mutations also showed identical species specificity in our study.

### Structural mutations revealed by PCR analysis

Among the 94 accessions, we were unable to obtain amplicons of *PSY1* N‐terminal, *PSY1* C‐terminal, *Lcyb* and *CCS* in 18, 19, 4 and 32 accessions respectively (Table S3). As we could not conduct SMRT sequencing using accessions showing abnormal patterns of amplification, we performed PCR using modified conditions to supplement the SMRT sequencing results. These modifications included the use of longer extension times (up to 10 min) and alternate primers that bind to other regions of the target genes, such as promoter regions. In the last two genes, *PSY2* and *ZEP*, amplicons were normally amplified in the whole accessions.

#### Structural mutation of *PSY1*

No amplicons for *PSY1* N‐terminal SMRT sequencing were obtained for 18 accessions, and an amplicon of a much larger size (3 kb) than expected was obtained for one *C. baccatum* accession (ACN 64). For *PSY1* C‐terminal region, no amplicons were obtained for 19 accessions, and an amplicon with a much larger size was also produced for ACN 64; for another *C. baccatum* accession (ACN 69), an amplicon was only obtained for the N‐terminal region (Figure S3a). PCR analysis revealed a 1395 bp insertion in the third intron in ACN 64 (*psy1‐in1*). As this insertion was located between the binding site of the first round reverse primer and that of the second round forward primer used for library construction, larger than expected amplicons were obtained for both the N‐ and C‐terminal regions of *PSY1*. In ACN 69, we successfully obtained an amplicon by long‐range PCR with an extension time of 10 min, revealing a 7 kb insertion in *PSY1* (*psy1‐in2*). However, we were not able to detect mutations in 18 accessions due to the lack of amplification of *PSY1*. Perhaps a huge insertion or deletion is present in *PSY1* in these accessions.

#### Structural mutation of *Lcyb*

We failed to obtain amplicons for *Lcyb* by SMRT sequencing from four *C. annuum* accessions (ACN 4, 15, 53 and 58). As the fruits of these accessions are deep orange, orange, yellow and light yellow, respectively, this issue is not significantly correlated with fruit colour. We did not obtain any clue about this structural mutation of *Lcyb* from previous studies. Perhaps mutations that impede the PCR amplification of this region may exist near the primer binding sites.

#### Structural mutation of *CrtZ‐2*

Among amplification products of the N‐terminal region of *CrtZ‐2* used for library construction, 19 accessions produced amplicons of aberrant sizes (Table S3). In 17 accessions, a 960 bp insertion (*crtz‐in*) was detected at the first intron. All accessions with this mutation are *C. annuum* lines. Accessions possessing this mutation show a wide range of fruit colours from light yellow to red. In two accessions (ACN 27 and 69), we detected 84 bp deletion at the first intron region (Figure S3b); these accessions are *C. annuum* and *C. baccatum* respectively (Table S6).

#### Structural mutation of *CCS*

No amplicons of *CCS* were obtained for 32 accessions via SMRT sequencing (Table S3). To examine the structural variation in *CCS* in these accessions, we designed a forward primer based on a −4.3 kb upstream region of the start codon. In wild‐type red peppers, a 5.9 kb amplicon is expected to be produced when this forward primer and the reverse primer used for the first library construction are employed in PCR. For 23 accessions, 1 kb amplicons were obtained (*ccs‐p1*). Sequence analysis revealed a 4879 bp deletion from −3686 nt upstream of start codon to 1193 nt downstream of the start codon. All accessions with this deletion are *C. annuum* lines (Table S6). We obtained 2.5 kb amplicons (*ccs‐p2*) for five *C. baccatum* accessions (ACN 64, 66, 67, 68 and 69). Sequencing analysis revealed a 2968 bp deletion from −3010 to −42 bp upstream of the start codon in these accessions. We also detected structural variation in the promoter regions of nine accessions. As the primer binding sites of these accessions were intact, libraries were constructed and sequenced as usual. However, during PCR to target the promoter region, larger than expected bands (6 kb) were produced (*ccs‐p3*). We detected many indel mutations, including a 11 bp deletion, 197 bp deletion and 5 bp insertion in *C. annuum* (ACN 12)*, C. chinense* (ACN 75, 78, 79, 80, 81 and 82) and *C. frutescens* (ACN 90 and 91) accessions. After detecting structural variation in the promoter region of *CCS*, we performed PCR using the same primer and a greatly extended elongation time (10 min). As a result, an almost 9 kb amplicon was obtained from one *C. annuum* accession (ACN 42). Upon further analysis, a 3 kb insertion at position 898 nt of the coding region was discovered (Figures S4 and 4). However, we did not identify the mutations in three accessions, including one *C. annuum* (ACN 27), one *C. baccatum* (ACN 65) and one *C. pubescens* accession (ACN 93) after SMRT sequencing and promoter analysis by PCR. It may imply that other types of mutations are present in these accessions.

**Figure 4:**
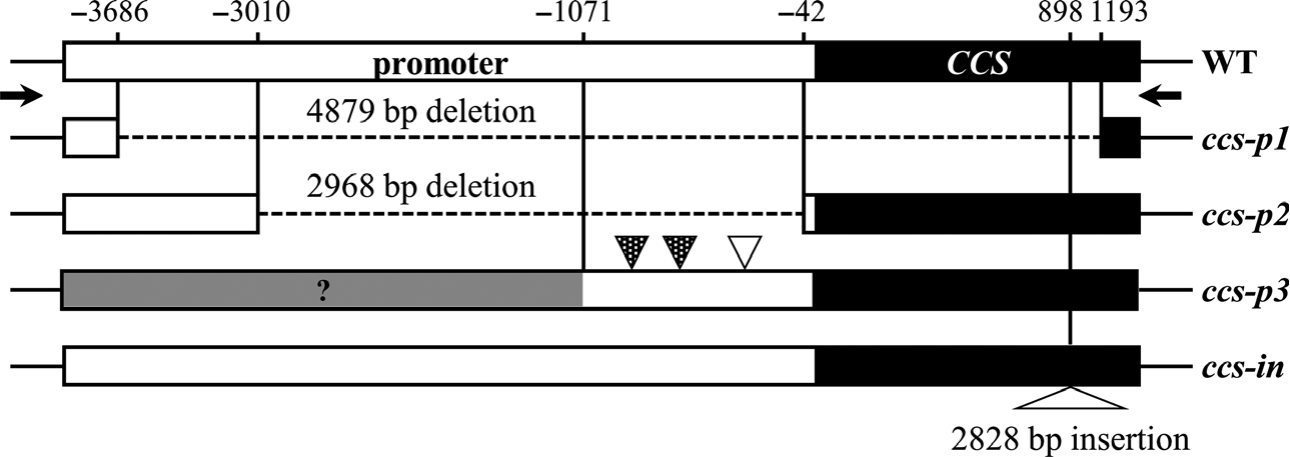
Structural mutations of capsanthin‐capsorubin synthase (*CCS*) in various *Capsicum* accessions. Schematic representation of the structural mutations in the promoter and coding regions of *CCS* in the non‐red fruit varieties. Empty and black rectangles indicate the promoter and coding region of *CCS*, respectively, and the grey rectangle with question mark represents the region whose sequence was not identified. *ccs‐p1* has a 4879 bp deletion from 3686 nt of the promoter region to 1193 nt of the coding region. *ccs‐p2* has a 2968 bp deletion from 3010 to 42 nt of the promoter region. *ccs‐p3* has several deletions and insertions. The *ccs‐in* allele contains a 2828 bp insertion in the coding region at 898 nt. Black‐patterned and empty triangles indicate a deletion and insertion respectively. Black arrows indicate primer locations [Correction added on 8 January 2019, after first online publication. Information regarding Figures 3a,b,d, and Figures 4 and 5 were previously incorrect and are now updated in this version.].

### Classification of carotenoid profiles

Among the 94 *Capsicum* accessions, we selected 43 accessions representing each different type of allelic variation in the candidate genes. For each sample, we performed carotenoid profiling via UPLC, with two independent replicates. We classified the 43 *Capsicum* accessions into four different groups based on chromatogram pattern (Figure S5 and Table S6). In group I (eight accessions), capsanthin is the major carotenoid, whereas no lutein was detected. In group II (26 accessions), all carotenoids except capsanthin were detected; the three most abundant carotenoids are lutein, antheraxanthin and violaxanthin. In group III (seven accessions), low levels of most carotenoids were detected. Although high levels of lutein were detected in this group, as in group II, cryptoxanthins and carotenes were not detected in group III. In group IV (two accessions), α‐cryptoxanthin and antheraxanthin are the major carotenoids, whereas no lutein or capsanthin was detected. In summary, accessions in group I have reddish fruit (pink to red) due to the accumulation of capsanthin. The orange‐like colour (yellow to orange) of fruits in group II accessions may be due to the presence of a combination of lutein, antherxanthin and violaxanthin. The yellowish colour (ivory to light yellow) of fruit in group III accessions may be due to the presence of lutein and basal levels of other carotenoids.

### Correlation between carotenoid levels and genotype

**Figure 5:**
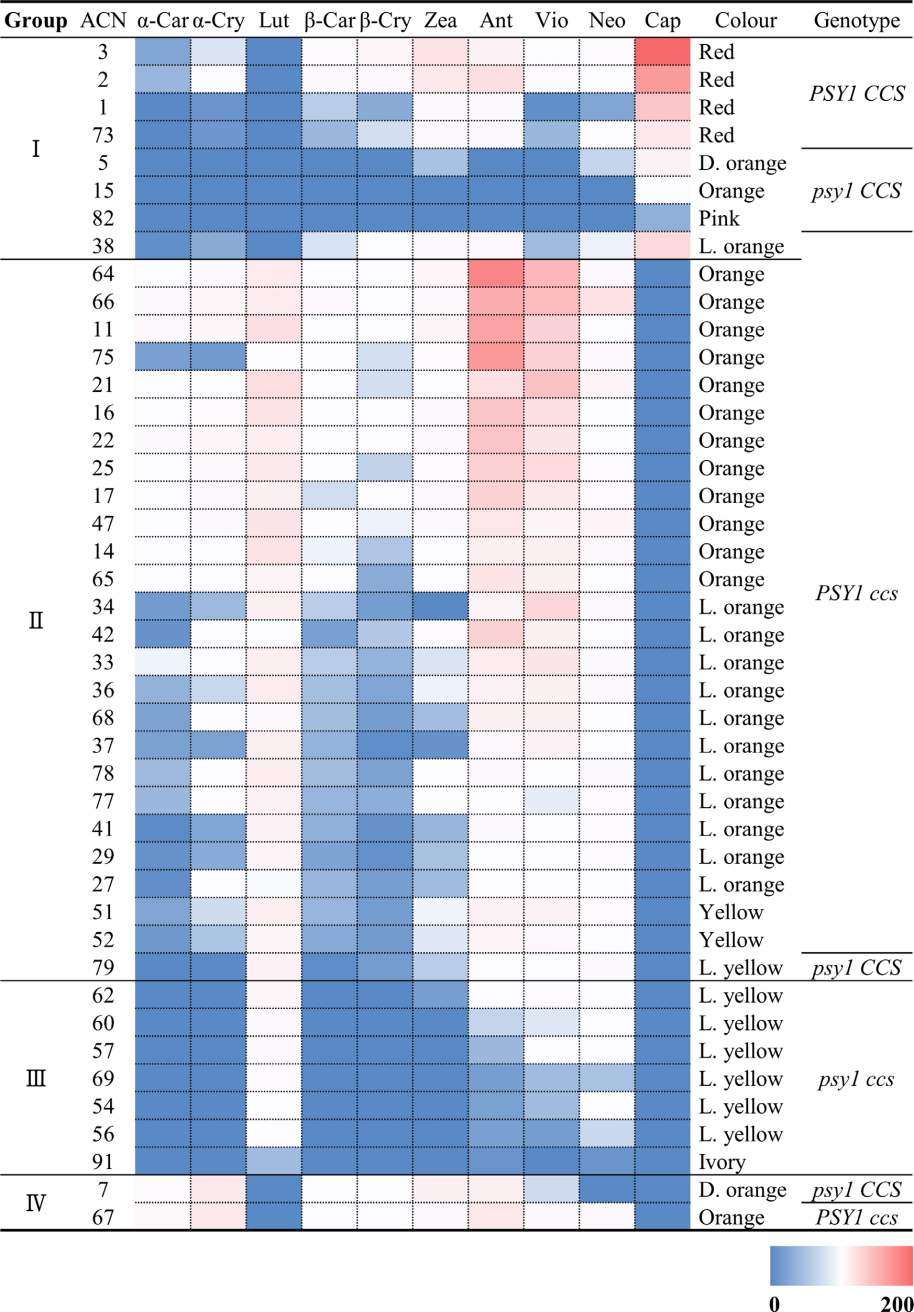
Heatmap of the levels of 10 carotenoids in 43 *Capsicum* accessions. The colour of each square indicates the absolute carotenoid content. The amount of each component is represented by the intensity of the blue (low) to red (high) colour, as depicted in the colour scale below. The group numbers of accessions in the chromatogram are listed in the leftmost column, and the genotypes represented by *PSY1* and capsanthin‐capsorubin synthase are described in the rightmost column. The name of each carotenoid is abbreviated (first three letters in its name). The chromatogram groups and genotypes are consistent, except for four accessions (ACN 38, 79, 7 and 67). [Correction added on 8 January 2019, after first online publication. Information regarding Figures 3a,b,d, and Figures 4 and 5 were previously incorrect and are now updated in this version.].

To investigate whether the carotenoid profile of each sample is correlated with the genotypes of the candidate genes, we first classified the knockout mutations of *PSY1*, *PSY2*, *Lcyb*, *CrtZ‐2*, *ZEP* and *CCS*, which are expected to abolish the functioning of these genes. Such mutations include non‐sense mutations and indels in coding regions causing a frame‐shift and huge deletions occurring in the promoter regions of these genes. Mis‐sense mutations and variations in intron were excluded in this classification. As critical mutations satisfying above criteria were only discovered in *PSY1* and *CCS*, we constructed a heatmap of carotenoid levels in 43 pepper accessions and used it for comparisons between the levels of these compounds and *PSY1* and *CCS* genotypes (Figure 5). In the heatmap, carotenoid levels are represented in blue (minimum), white (median) and red (maximum). All accessions in group I except for one *C. annuum* (ACN 38) accession have a functional *CCS*. Group I can be divided into two subgroups according to *PSY1* genotype. Accessions with a functional *PSY1* accumulate high levels of capsanthin, whereas those with a dysfunctional *PSY1* show reduced capsanthin levels and do not produce red fruit; the fruits in this subgroup are deep orange (ACN 5), orange (ACN 15) and pink (ACN 82). Accessions in group II have a functional *PSY1* (except for one *C. chinense* accession; ACN 79) and a mutated *CCS*. Their fruit colours range from yellow to orange. In group III accessions, both *PSY1* and *CCS* are impaired, and the fruits are ivory to light yellow. Finally, one *C. annuum* (ACN 7) and one *C. baccatum* accession (ACN 67) in group IV have a *psy1/CCS* and *PSY1/ccs* genotype respectively. It is difficult to correlate the carotenoid profiles of these two accessions with their genotypes. In summary, chromatogram groups I to III are correlated with the genotypes of *PSY1* and *CCS*, except for two accessions (ACN 38 and 79).

## Discussion

Peppers display diverse fruit colours ranging from ivory to red due to the levels of various carotenoid pigments. However, the genetic variation in carotenoid biosynthetic genes does not fully explain these differences in fruit colour. In this study, we identified diverse allelic variations in *PSY1*, *PSY2*, *Lcyb*, *CrtZ‐2*, *ZEP* and *CCS* for 94 *Capsicum* accessions via SMRT sequencing and identified the compositions and levels of carotenoids for 43 accessions via UPLC. Since the most of premature stop mutations we found were primarily focused on the two genes, *PSY1* and *CCS*, genotypic groups were divided on the basis of both genes. We were able to show clear correlations between carotenoid profiles and allelic variations represented by various combinations of *PSY1* and *CCS*.

Recent progress in genome sequencing technologies has enabled the analysis of specific regions of the genome as well as entire genomes. The use of targeted enrichment methods considerably reduces the amount of data required for analysis and the complexity of analysis. Furthermore, targeted sequencing results in increased read depth, thereby improving sequencing accuracy (Jupe *et al*., 2013). SMRT sequencing is an effective approach for targeted sequencing because it yields longer read lengths than standard sequencing techniques. The use of long reads allows for successful sequencing and assembly, even when a subset of the genome have high copy numbers and highly repetitive sequences (Rhoads and Au, 2015). The utility of SMRT sequencing has recently been expanded, as it can be combined with other approaches. For example Witek *et al*. (2016) combined resistance gene enrichment sequencing (RenSeq) with SMRT sequencing to clone a late blight‐resistance gene in potato. The use of SMRT sequencing accelerated the assembly of a full‐length nucleotide binding‐site leucine‐rich repeat (NB‐LRR) gene with a high copy number that is highly homologous to other sequences. In this study, we demonstrated that targeted‐SMRT sequencing of candidate carotenoid biosynthetic genes could be utilized to identify allelic variations in six such genes. A similar approach could be used to investigate genes controlling other secondary metabolites whose biosynthetic pathways and associated enzymes are well elucidated.

To validate the accuracy of the SMRT sequencing data, we compared the allelic variations detected in this study with those of previous studies. Although most variations detected in this study are novel, some are identical to previously reported alleles, such as *ccs‐c3, ccs‐3* and *ccs‐c11* (Figure 2b). These results demonstrate that our SMRT analysis successfully identified novel allelic variations. Kilcrease *et al*. (2015) found that *C. annuum* ‘Costeño Red’ contains several mutations in *CCS*, including G235C and C630T. We also detected these mutations by SMRT sequencing in red fruit accessions ACN 64, 72, 73 and 74 (data not shown). In 32 accessions, however, we failed to obtain amplicons of *CCS* for SMRT library construction (Table S3). Indeed, many researchers have failed to identify *CCS* in non‐red pepper varieties, raising the possibility of a deletion in *CCS* (Bouvier *et al*., 1994; Lang *et al*., 2004; Lefebvre *et al*., 1998; Popovsky and Paran, 2000). In this study, we identified three types of structural variation in the promoter region of *CCS* by PCR analysis. Furthermore, UPLC analysis showed that two of these three structural mutations resulted in no accumulation of capsanthin due to the dysfunction of *CCS*. For example, accessions with the *ccs‐p1* and *ccs‐p2* alleles are characterized by the absence of capsanthin (Table S6). In contrast, a very small amount of capsanthin (0.59 mg/100 g dry weight) was detected in one *C. chinense* ACN 82 harbouring *ccs‐p3*. It appears that the mutation in the promoter region of *CCS* in ACN 82 does not fully abolish the expression of this gene, allowing for minimal levels of expression, which leads to the pink fruit colour observed in this accession. In addition to these mutations in the promoter region, a structural mutation in the coding region was also detected in one *C. annuum* accession (ACN 42); the *ccs‐in* allele might result in dysfunctional *CCS*, as no capsanthin was detected in ACN 42. However, we failed to obtain *CCS* amplicons by PCR analysis in three accessions, including one *C. annuum* (ACN 27), one *C. baccatum* (ACN 65) and one *C. pubescens* accession (ACN 93).

We were unable to obtain amplicons from *PSY1* for SMRT sequencing in 18 accessions, possibly due to structural variation. Moreover, we obtained unexpected patterns of amplification for two *C. baccatum* accessions (ACN 64 and 69; Table S3). Although we could not identify the mutations in 18 accessions, we detected two insertional mutations in the latter two accessions. The *psy1‐in1* allele of *C. baccatum* ACN 64 contains a 1.4 kb insertion in the third intron, and the *psy1‐in2* allele of *C. baccatum* ACN 69 contains a ~7 kb insertion at the C‐terminal region (exact position remains to be uncovered). Even though we were unable to confirm the expression level of *PSY1*, the insertion in the *psy1‐in1* allele might not influence the expression of this gene, since the carotenoid profile of ACN 64 indicates that *PSY1* is functional. On the contrary, the carotenoid profile of ACN 69 shows that the function of *PSY1* is impaired in this accession (Table S6).

The three‐locus model has been proposed to explain fruit colour in *Capsicum* (Hurtado‐Hernandez and Smith, 1985). According to this model, eight different combinations of these loci determine fruit colour. In this study, we classified the results in the chromatogram based on the composition and amount of each carotenoid and correlated the results to the genotypes of carotenoid biosynthetic genes to explain pepper fruit colour for the first time (Figure 5). We analysed the six carotenoid biosynthetic genes, but only *PSY1* and *CCS* appeared to contribute to fruit colour. In other words, since the majority of mutations in the rest four genes were mis‐sense mutations, and the number of accessions bearing such mutations was very small, classification of genotypes using those genes were virtually restricted. Overall, the chromatogram groups and genotypes represented by various combinations of *PSY1* and *CCS* corresponded with each other. However, for accessions in group II, the carotenoid profiles were subdivided into two classes based on the amounts of specific carotenoid components. In one subgroup (ACN 64 to ACN 65 in Figure 5; 12 accessions), moderate levels of carotene, cryptoxanthin and zeaxanthin are present and the fruits are orange. In the other subgroup (ACN 34 to ACN 52 in Figure 5; 13 accessions), these three carotenoids were almost undetectable, and the fruits are light orange or yellow. The reason for this separation could be explained by the presence of a gene with unknown function. We postulate that this gene might be *C1*, the last locus of the three‐locus model, whose identity has not yet been determined.

Noticeably, ACN 5, 15 and 82 (belonging to group I) have non‐red fruit despite the presence of capsanthin. Considering that capsanthin is the major carotenoid in red pepper, these samples were also expected to have red fruit. However, their fruit colour varies from deep orange to pink. The reason for this variation can be explained by the concept of dilution. Papaya mainly contains lycopene (more than 60% of total carotenoids), although the absolute content of this compound is much lower in papaya than in tomato, which has a very high lycopene content. Despite the accumulation of lycopene, papaya fruit is orange (Khachik *et al*., 1). Interestingly, one *C. annuum* ACN 38 contains capsanthin despite the non‐sense mutation of *CCS* (*ccs‐c9*). Considering that capsanthin does not generally accumulate in *CCS* knockout plants (Figure 5 and Table S6), perhaps this accession could serve as a valuable resource for identifying an additional *CCS* gene in the future.

*PSY1* is a key gene required for carotenoid biosynthesis (Fraser *et al*., 2007). *PSY1* was previously identified as a candidate gene for orange fruit colour (Huh *et al*., 2001). In orange‐coloured *C. chinense* Habanero, *PSY1* contains a point mutation at the splicing acceptor region (Kim *et al*., 2010). These findings demonstrate that *PSY1* plays a key role in determining fruit colour in pepper. In this study, we detected knockout mutations in *PSY1* in pepper accessions with non‐red fruits (Figure 2a). Since PSY1 catalyses the first‐committed step of carotenoid biosynthesis, *PSY1*‐knockout plant cannot synthesize carotenoids. However, carotenoids were indeed synthesized in 12 *PSY1*‐knockout accessions, although their total carotenoid levels were much lower than those of other accessions with functional *PSY1* (Figure 5). These results point to the existence of another gene whose function can substitute for that of *PSY1*. Actually, tomato contains two additional *PSY* genes: *PSY2* and *PSY3*. Giorio *et al*. (2008) reported that transcripts of both *PSY1* and *PSY2* were detectable in various tomato tissues including leaf, sepal, petal and even fruit although the expression level of *PSY1* in fruit was significantly higher than that of *PSY2*. Kachanovsky *et al*. (2012) proposed that *PSY3* might function in roots under stress conditions similar to that in cereal crops. However, it is difficult to imagine that the functions of *PSY* homologs can substitute for that of *PSY1*, as the silencing of *PSY2* and *PSY3* in mature tomato fruit did not result in definite phenotypic changes (Fantini *et al*., 2013). The pepper genome contains two putative *PSY* genes whose functions have not yet been clearly elucidated. On the basis of the previous reports about tomato *PSY2*, we decided to analyse pepper *PSY2* along with *PSY1* and revealed that sequence of *PSY2* was very well conserved among *Capsicum* accessions without noticeable mutations in protein. In other words, every detected mutations were mis‐sense mutations, which indicates the importance of *PSY2* in carotenoid biosynthesis. It appears that *PSY1*‐knockout plant could synthesize basal level of carotenoid due to the intact function of *PSY2*. Nevertheless, more functional studies are needed to show the involvement of *PSY2* in fruit coloration in *Capsicum*.

Two unidentified carotenoid pigments were detected in the chromatograms of non‐red pepper accessions (Figure S5). The retention times of these carotenoids were approximately 6.4 and 7.2 min respectively. As we only analysed well‐known carotenoids due to the unavailability of whole carotenoid standards, other carotenoids whose identities have not been well studied might have been detected. Indeed, the orange‐coloured compound cucurbitaxanthin A, also known as capsolutein, was previously identified in red pepper (Hornero‐Méndez and Mínguez‐Mosquera, 1998). According to Hornero‐Méndez *et al*. (2000), cucurbitaxanthin A is derived from antheraxanthin, a substrate of CCS. Since unknown carotenoids have only been discovered in peppers with impaired *CCS*, we hypothesize that antheraxanthin might be converted into cucurbitaxanthin A instead of capsanthin in these accessions. Also, the transition of antheraxanthin into cucurbitaxanthin A is likely not a frequent reaction in peppers with active CCS. More studies are needed to reveal the identities of these compounds and their functions in coloration.

In conclusion, we demonstrated that target gene analysis using SMRT sequencing is an efficient method for identifying genetic variations in carotenoid biosynthetic genes controlling fruit colour. Total 12, 3, 7, 9, 16 and 10 novel alleles with mutated coding region of *PSY1*, *PSY2*, *Lcyb*, *CrtZ‐2*, *ZEP* and *CCS* were identified in this study. Such allelic variations could be useful for developing functional molecular markers to predict fruit colours and to further investigate carotenoid biosynthesis in pepper.

## Experimental procedures

### Plant materials

A total of 94 *Capsicum* accessions from *C. annuum, C. baccatum, C. chacoense, C. chinense, C. eximium, C. frutescens, C. praetermissum* and *C. pubescens* were used in this study. These accessions were provided by the National Agrobiodiversity Center (Jeonju, Korea). Most of the accessions were *C. annuum*, comprising 62 accessions (Table S1). All plants were grown in an open field (SNU, Suwon, Korea). *Capsicum* accessions were selected to represent eight different types of accessions using a QPcard 203 to analyse the colour of fruit at the mature stage: red, deep orange (D. orange), orange, light orange (L. orange), yellow, light yellow (L. yellow), ivory and pink (Figures S1 and S2). Among the 94 accessions, 14 accessions had red fruits and 7, 22, 23, 12, 12, 2 and 2 accessions had deep orange, orange, light orange, yellow, light yellow, ivory and pink fruit respectively.

### Library construction for SMRT sequencing

PCR was performed using Ex‐taq (Takara, Shiga, Japan) with 100 ng of each DNA template and 10 pmol of gene‐specific primers for *PSY1*, *PSY2*, *Lcyb*, *CrtZ‐2, ZEP* and *CCS*. To ensure a similar sequencing depth, primers were designed to produce amplicons of similar sizes (Table S2). Two‐step PCR was conducted for library construction (Figure 1). The first PCR was performed to amplify regions of the target genes using the first set of PCR primers. The first set of primers was designed to include a common sequence for annealing in the second PCR along with the gene‐specific region. The PCR conditions were as follow: 95 °C for 4 min, followed by 35 cycles of 95 °C for 30 s, 55 °C for 30 s and 72 °C for 1 min 40 s. The second PCR was performed to amplify the PCR product from the first PCR using the second set of PCR primers. The second set of primers was designed to include barcode sequences to identify each samples and a common sequence for annealing in the second PCR. The PCR conditions were as follow: 95 °C for 4 min, followed by 30 cycles of 95 °C for 30 s, 60 °C for 30 s and 72 °C for 1 min 50 s. After two‐step PCR, each PCR amplicon was purified using a QiaQuick PCR Purification Kit (Qiagen, Hilden, Germany) and quantified by NanoDrop (Take3; Biotek, Winooski, Vermont). All amplicons were pooled in a single tube for SMRT sequencing using equal quantities of each molecule. The required volume of each amplicon was calculated according to DNA concentration.

### SMRT sequencing

Single‐molecule real‐time sequencing was performed in two different ways to analyse the sequence of six carotenoid biosynthetic genes. In case of *PSY1*, *LcyB*, *CrtZ‐2* and *CCS*, sequencing was performed according to the P5‐C3 chemistry (Macrogen, Seoul, Korea). The rest two genes, *PSY2* and *ZEP*, sequencing was performed according to the P6‐C4 chemistry (DNA Link, Seoul, Korea). The SMRT bell library was constructed using end‐repair, ligation and exonuclease purification strategies. SMRT bell templates were bound to polymerase molecules for 4 h at 25 °C and then resulting polymerase‐template complexes were immobilized on nanofabricated SMRT cells containing an array of zero‐mode waveguides (ZMWs), which were analysed by sequencing to generate reads.

### Sequencing data analysis

Data from SMRT sequencing were analysed using SMRT analysis software v2.3.0. Raw reads were filtered, demultiplexed by barcode sequence and aligned to six reference genomic sequences with the default option using the RS_Resequencing_Barcode.1 protocol from SMRT Portal. The cmp.h5 file from the alignment was split by sample, and variants were called using the Quiver algorithm. Variants with a minimum coverage of 20 and minimum confidence of 20 were used for further analysis. The consensus sequence of each gene was extracted based on the reference genomic sequence and variants using Quiver.

### Carotenoid extraction and saponification

Pericarp tissue samples without placenta were collected from fruits at the mature stage. The carotenoid pigments were extracted according to method of Collera‐Zúñiga *et al*. (2005) but using different volumes of chemicals. Acetone (20 mL) was added to 1 g of freeze‐dried pericarp and incubated at 4 °C for 20 h to extract the colour. The extracts were evaporated to dryness and incubated with 3 mL acetone, 3 mL methanol and 1 mL 30% potassium hydroxide/methanol at room temperature for 2 h 30 min in the dark. After saponification, the extracts were transferred to a separatory funnel with 20 mL diethyl ether, shaken and left to settle. The extracts were combined with 2 mL of 10% NaCl to separate the phases and to transfer the pigments to the ether. Subsequently, 2 mL of 2% Na_2_SO_4_ was added to remove all water in the ether phase. The ether phase was collected and vacuum‐dried. The resulting dry residue was dissolved in 2 mL acetone and filtered through an Acrodisc syringe filter (13 mm, 0.2 μm; Pall Corporation, New York, NY).

### Carotenoid analysis by UPLC

Carotenoids were analysed using an Acquity UPLC‐H‐Class system (Waters, Milford, MA). Separation was performed using an Acquity UPLC HSS T3 column (2.1 × 100, 1.8 μm) at 35 °C. The mobile phase was a binary solvent system consisting of phase A (acetonitrile/methanol/methylene chloride, 65/25/10, v/v/v) and phase B (distilled water). The gradients were programmed as previously described (Kim *et al*., 2016). The UV wavelength was set to 450 nm. For qualitative and quantitative analyses of carotenoids, 10 standards were purchased from Sigma‐Aldrich (St. Louis, MO), including antheraxanthin, capsanthin, lutein, neoxanthin, violaxanthin, zeaxanthin, α‐carotene, α‐cryptoxanthin, β‐carotene and β‐cryptoxanthin.

## Conflict of interest

The authors declare no conflict of interest.

## Supporting information


**Figure S1** Fruit colour index used in this study.
**Figure S2** Mature fruits of the pepper accessions used in this study.
**Figure S3** Amplicons of *PSY1* and *CrtZ‐2* with unexpected sizes.
**Figure S4** Amplicons of capsanthin‐capsorubin synthase with unexpected sizes.
**Figure S5** Chromatograms of carotenoids used for group classification.
**Table S1** Classification of the species and fruit colours of the *Capsicum* accessions analysed in this study.
**Table S2** Primers used in this study.
**Table S3** Details about library construction.
**Table S4** Summary of single‐molecule real‐time sequencing results.
**Table S5** Number of mutations in non‐red fruit accessions discovered by single‐molecule real‐time sequencing.

**Table S6** Carotenoid profiles and genotypes.
