# Supplementary material for: Single‐molecule real‐time sequencing reveals diverse allelic variations in carotenoid biosynthetic genes in pepper (Capsicum spp.)
Source: Plant Biotechnol J. 2018 Dec 9;17(6):1081–93. doi: 10.1111/pbi.13039 (PMC6523600; doi:10.1111/pbi.13039)
Supplement: Supplementary file 1 — Figure S1 Fruit colour index used in this study. Figure S2 Mature fruits of the pepper accessions used in this study. Figure S3 Amplicons of PSY1 and CrtZ‐2 with unexpected sizes. Figure S4 Amplicons of capsanthin‐capsorubin synthase with unexpected sizes. Figure S5 Chromatograms of carotenoids used for group classification. Table S1 Classification of the species and fruit colours of the Capsicum accessions analysed in this study. Table S2 Primers used in this study. Table S3 Details about library construction. Table S4 Summary of single‐molecule real‐time sequencing results. Table S5 Number of mutations in non‐red fruit accessions discovered by single‐molecule real‐time sequencing. [file PBI-17-1081-s002.docx]

**Supporting information**

**Table S1.** Classification of the species and fruit colors of the *Capsicum* accessions analyzed in this study.

| Species | Fruit color | | | | | | | | |
| --- | --- | --- | --- | --- | --- | --- | --- | --- | --- |
|  | Red | D. orange | Orange | L. orange | Yellow | L. yellow | Ivory | Pink | Total |
| *C. annuum* | 3 | 7 | 16 | 16 | 11 | 9 | 0 | 0 | 62 |
| *C. chinense* | 2 | 0 | 4 | 1 | 0 | 1 | 0 | 0 | 8 |
| *C. baccatum* | 2 | 0 | 0 | 0 | 0 | 0 | 0 | 0 | 2 |
| *C. frutescens* | 3 | 0 | 1 | 3 | 0 | 2 | 0 | 2 | 11 |
| *C. chacoense* | 1 | 0 | 0 | 3 | 0 | 0 | 2 | 0 | 6 |
| *C. pubescens* | 1 | 0 | 0 | 0 | 0 | 0 | 0 | 0 | 1 |
| *C. praetermissum* | 0 | 0 | 1 | 0 | 1 | 0 | 0 | 0 | 2 |
| *C. eximium* | 2 | 0 | 0 | 0 | 0 | 0 | 0 | 0 | 2 |
| Total | 14 | 7 | 22 | 23 | 12 | 12 | 2 | 2 | 94 |

**Table S2.** Primers used in this study.

| **Name** | **Sequence (5’→3’)** | **Target gene** |
| --- | --- | --- |
| PSY1-P1-pacbio-F | TCGTCGGCAGCGTCGAGGTCGCTATAGGAGCCGA | *N-PSY1* |
| PSY1-P1-pacbio-R | GTCTCGTGGGCTCGGAACCCAACCGTACCAGCAAC | *N-PSY1* |
| PSY1-P2-pacbio-F | TCGTCGGCAGCGTCCTGTTTCACAGTTTTGCGAACTC | *C-PSY1* |
| PSY1-P2-pacbio-R | GTCTCGTGGGCTCGGTATTGGCTTCATTGGCCTTG | *C-PSY1* |
| PSY2-P1-pacbio-F | TCGTCGGCAGCGTCTGGGATAAACTAGGCTGAGGTG | *N-PSY2* |
| PSY2-P1-pacbio-R | GTCTCGTGGGCTCGGCAACTGGAAATCTGGAAACG | *N-PSY2* |
| PSY2-P2-pacbio-F | TCGTCGGCAGCGTCGGTGCAGGAGAACTGATGAG | *C-PSY2* |
| PSY2-P2-pacbio-R | GTCTCGTGGGCTCGGCATTCATGTCTTTGTTAGTGAAGA | *C-PSY2* |
| LCYB-pacbio-F | TCGTCGGCAGCGTCAGGACCCCATTTGCTGTTTT | *Lcyb* |
| LCYB-pacbio-R | GTCTCGTGGGCTCGGTCCGATCATTCTCCCGAGTT | *Lcyb* |
| CRTZ2-P1-pacbio-F | TCGTCGGCAGCGTCCCTGTGCATCAAGTCCTATTCA | *N-CrtZ-2* |
| CRTZ2-P1-pacbio-R | GTCTCGTGGGCTCGGATGAGAACACGTACAGCGCC | *N-CrtZ-2* |
| CRTZ2-P2-pacbio-F | TCGTCGGCAGCGTCGCCCTAACCTTTTTCCCAAA | *C-CrtZ-2* |
| CRTZ2-P2-pacbio-R | GTCTCGTGGGCTCGGGCTGCTTTTTCGTGGTGTTC | *C-CrtZ-2* |
| ZEP-P1-pacbio-F | TCGTCGGCAGCGTCTCCTTTCACTTCCTTTGGCCT | *N-ZEP* |
| ZEP-P1-pacbio-R | GTCTCGTGGGCTCGGAGCTTCACTGTGTCCGAACA | *N-ZEP* |
| ZEP-P2-pacbio-F | TCGTCGGCAGCGTCCAAACATTTCCGTTGTGTTG | *M-ZEP* |
| ZEP-P2-pacbio-R | GTCTCGTGGGCTCGGCAAACCACAGGATATCAACTTCC | *M-ZEP* |
| ZEP-P3-pacbio-F | TCGTCGGCAGCGTCGGACTTGGGAATGCCTCTAATG | *C-ZEP* |
| ZEP-P3-pacbio-R | GTCTCGTGGGCTCGGATGCTGTACAAATTTCCCGTTT | *C-ZEP* |
| CCS-pacbio-F | TCGTCGGCAGCGTCTGATTCCCCTAGTTCGGTATTTC | *CCS* |
| CCS-pacbio-R | GTCTCGTGGGCTCGGGCTTTTGTTTCACTTTTGCATTG | *CCS* |
| CDF4000 | CCCCTGGCACACCACTCTTGGAGCC | *CCS* |

Template-specific sequences are underlined.

**Table S3.** Details about library construction.

| **Amplicon** | *N-*  *PSY1* | *C-*  *PSY1* | *N-*  *PSY2* | *C-*  *PSY2* | *Lcyb* | *N-*  *CrtZ-2* | *C-*  *CtrZ-2* | *N-*  *ZEP* | *M-*  *ZEP* | *C-*  *ZEP* | *CCS* |
| --- | --- | --- | --- | --- | --- | --- | --- | --- | --- | --- | --- |
| # of samples with  the expected amplicon size | 75 | 74 | 94 | 94 | 90 | 75 | 94 | 94 | 94 | 94 | 62 |
| # of samples with  an unexpected amplicon size | 1 | 1 | 0 | 0 | 0 | 19 | 0 | 0 | 0 | 0 | 0 |
| # of samples with  no amplicon | 18 | 19 | 0 | 0 | 4 | 0 | 0 | 0 | 0 | 0 | 32 |

**Table S4.** Summary of SMRT sequencing results.

| P5-C3 chemistry | Raw | Filtered | Mapped |
| --- | --- | --- | --- |
| # of reads | 300,584 | 94,318 | 86,720 |
| # of read bases (bp) | 1,944,296,659 | 1,278,193,633 | 1,041,247,040 |
| Mean read length | 6,468 | 13,551 | 12,007 |
| # of sub-reads |  | 765,944 | 622,385 |
| # or sub-read bases (bp) |  | 1,247,348,883 | 782,702,782 |
| Mean sub-read length (bp) |  | 1,628 | 1,258 |

| P6-C4 chemistry | Raw | Filtered | Mapped |
| --- | --- | --- | --- |
| # of reads | 150,292 | 108,181 | 105,138 |
| # of read bases (bp) | 2,139,370,028 | 2,041,520,976 | 1,994,371,056 |
| Mean read length | 14,234 | 18,871 | 17,954 |
| # of sub-reads |  | 1,140,510 | 1,045,281 |
| # or sub-read bases (bp) |  | 1,994,371,056 | 1,524,371,035 |
| Mean sub-read length (bp) |  | 1,749 | 1,458 |

**Table S5.** Number of mutations in non-red fruit accessions discovered by SMRT sequencing.

| **Gene** |  | Exon |  |  |  | Intron |  |
| --- | --- | --- | --- | --- | --- | --- | --- |
|  | Total | SNP | Indel |  | Total | SNP | Indel |
| *PSY1* | 36 | 34 | 2 |  | 60 | 55 | 5 |
| *PSY2* | 7 | 7 | 0 |  | 20 | 12 | 8 |
| *Lcyb* | 31 | 29 | 2 |  | 0 | 0 | 0 |
| *CrtZ-2* | 16 | 16 | 0 |  | 58 | 44 | 14 |
| *ZEP* | 30 | 30 | 0 |  | 61 | 50 | 11 |
| *CCS* | 29 | 23 | 6 |  | 0 | 0 | 0 |

**Table S6.** Carotenoid profiles and genotypes. (*Attached Excel file)

**
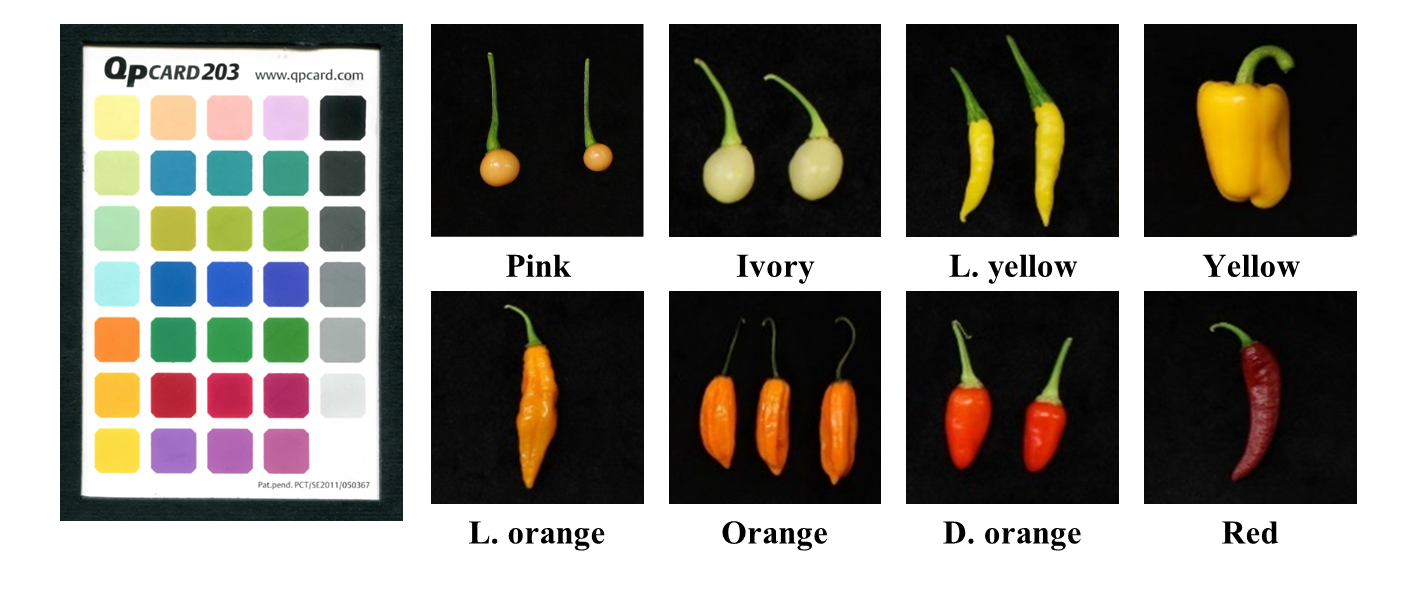
**

**Fig. S1.** Fruit color index used in this study

**
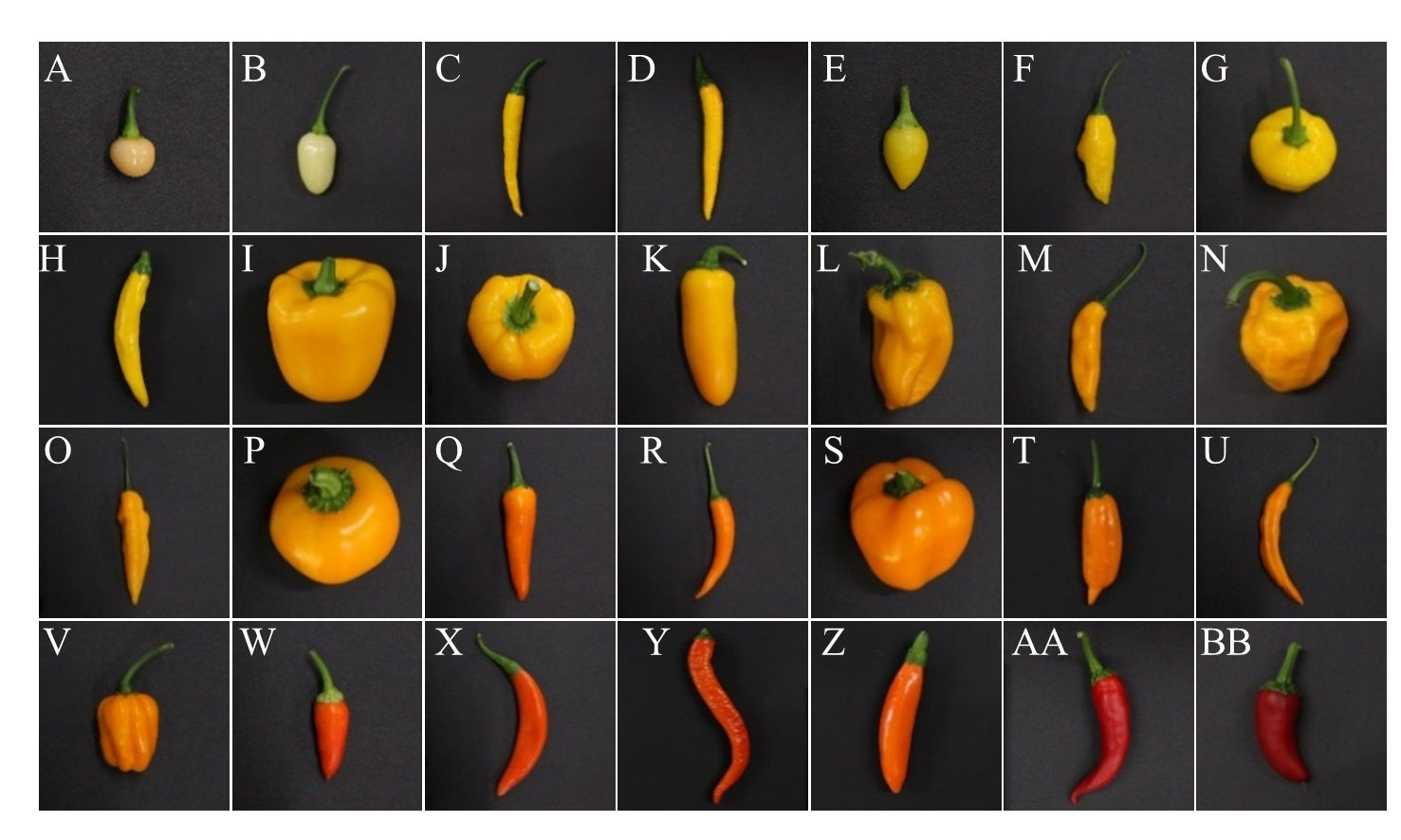
**

**Fig. S2.** Mature fruits of the pepper accessions used in this study.

The photographs show mature fruits from the following accessions: 82 (A), 91 (B), 54 (C), 69 (D), 58 (E), 79 (F), 80 (G), 61 (H), 51 (I), 52 (J), 53 (K), 30 (L), 68 (M), 31 (N), 77 (O), 42 (P), 14 (Q), 17 (R), 24 (S), 66 (T), 67 (U), 75 (V), 6 (W), 7 (X), 9 (Y), 10 (Z), 2 (AA), and 3 (BB).

**
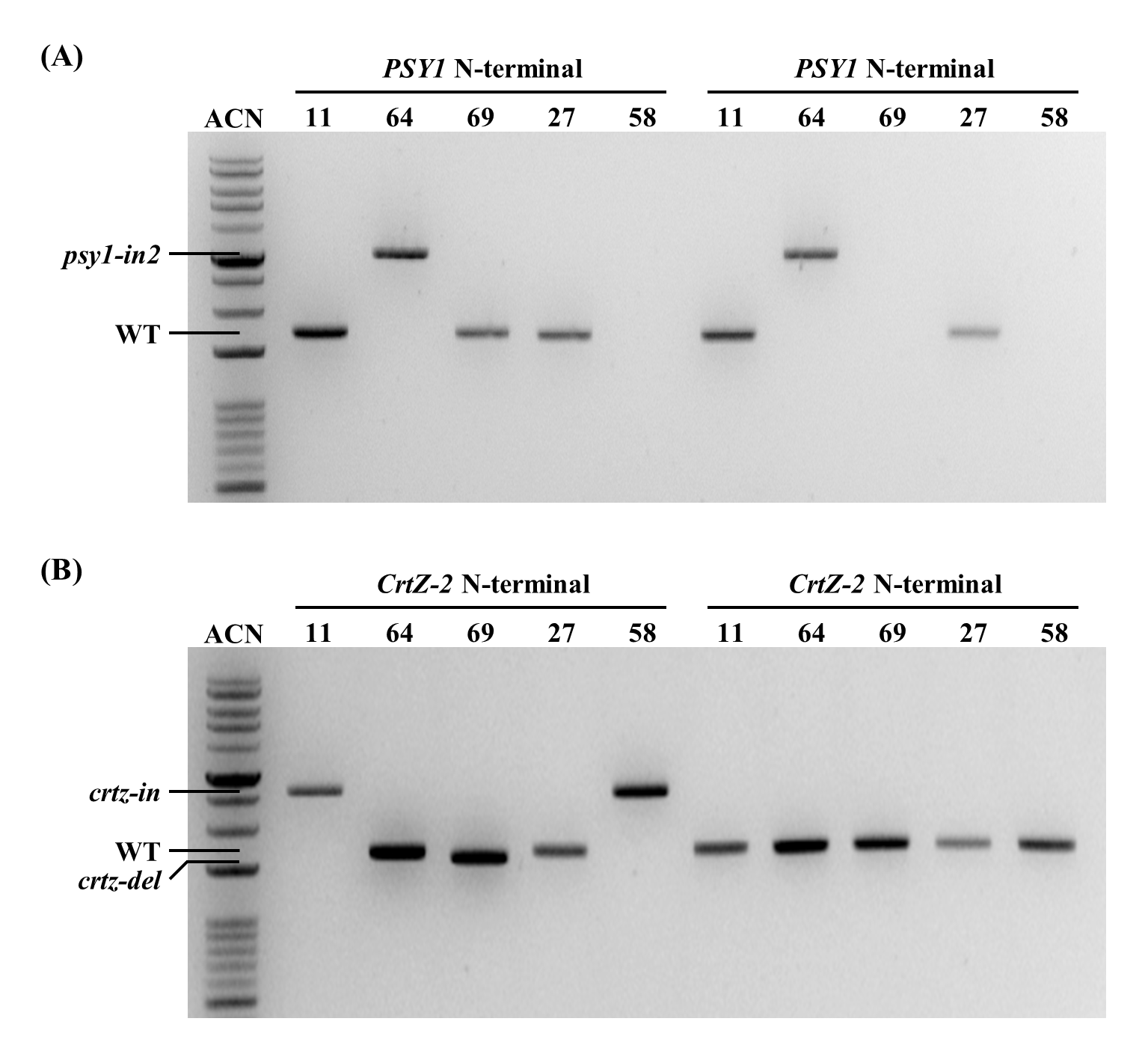
**

**Fig. S3.** Amplicons of *PSY1* and *CrtZ-2* with unexpected sizes.

Amplicons of the expected size were obtained from a red accession (ACN 27). (A) During *PSY1* library construction, larger than expected amplicons (above 3 kb) were obtained for both termini of ACN 64 (*psy-in1*). In ACN 69, only the N-terminal region was amplified. No amplicon was obtained for ACN 58. (B) During N-terminal amplification of *CrtZ-2*, large amplicons >2.5 kb (*crtz-in*) were obtained for ACN 11 and 58, whereas slightly smaller than expected amplicons (*crtz-del*) were detected in ACN 69. Every accession showed bands of the expected size for C-terminal amplification of *CrtZ-2*.

**
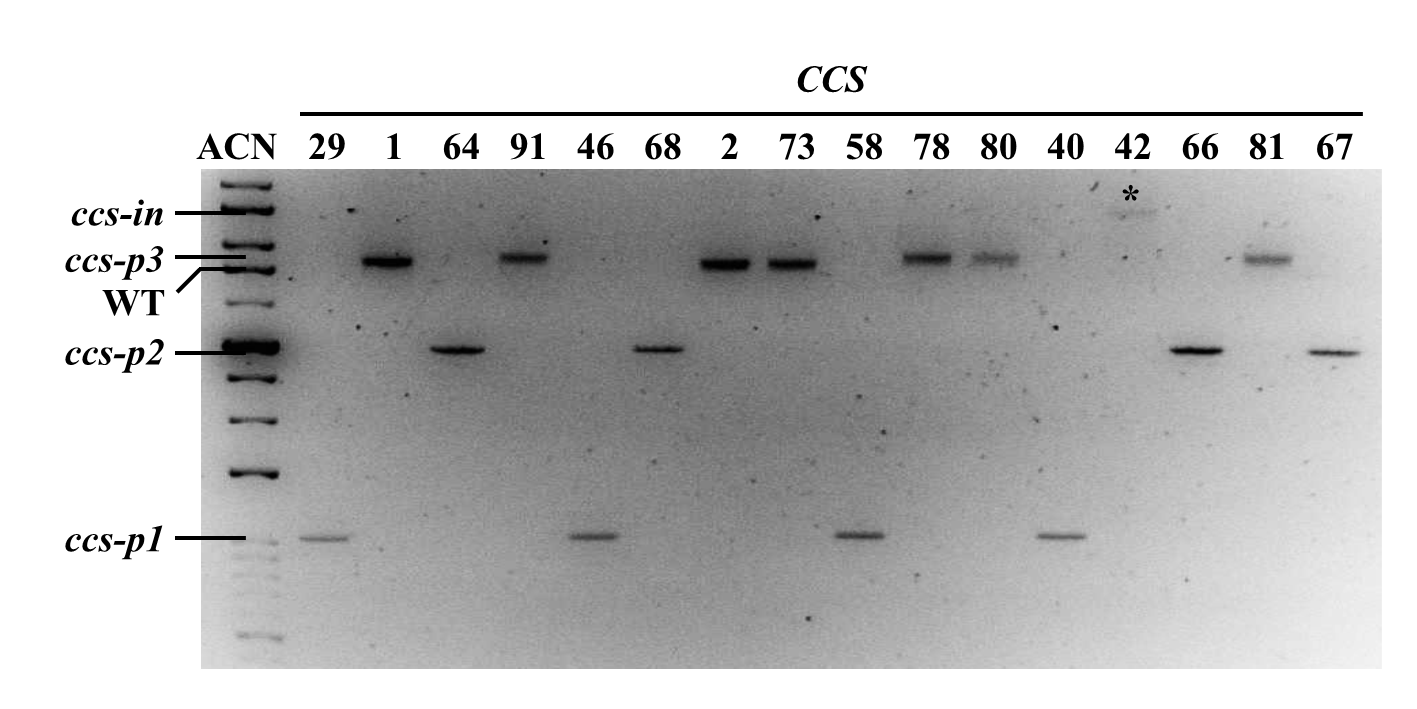
**

**Fig. S4.** Amplicons of *CCS* with unexpected sizes.

*CCS* amplicons of various sizes were obtained by PCR targeting the promoter region. Amplicons of the expected size were obtained from red accessions (ACN 2 and 73). Two types of deletions (*ccs-p1* and *ccs-p2*) and two types of insertions (*ccs-p3* and *ccs-in*) were detected. An asterisk indicates the presence of an amplicon with a lower brightness compared to amplicons from other accessions.

**
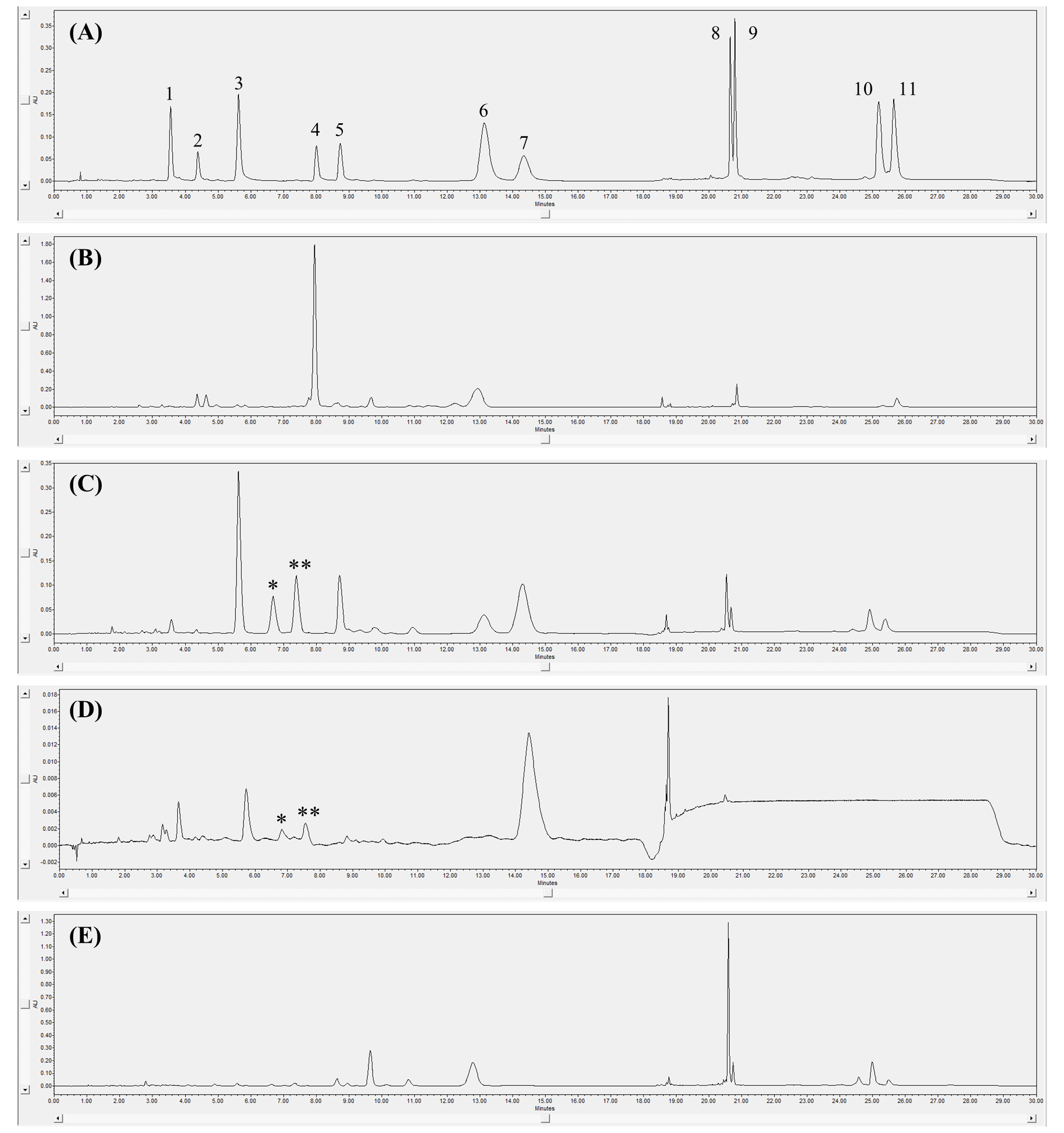
**

**Fig. S5.** Chromatograms of carotenoids used for group classification.

Each chromatogram shows a mixture of standard carotenoids (A) and carotenoids extracted from ACN 3 (B), ACN 16 (C), ACN 69 (D), and ACN 7 (E), belonging to group I, II, III, and IV, respectively. Each peak is numbered in order based on retention time: neoxanthin (1), capsorubin (2), violaxanthin (3), capsanthin (4), antheraxanthin (5), zeaxanthin (6), lutein (7), α-cryptoxantin (8), β-cryptoxanthin (9), α-carotene (10), and β-carotene (11). Asterisks indicate peaks of unknown carotenoids. The major carotenoid components of each group were capsanthin, violaxanthin, lutein, and β-cryptoxanthin, respectively. Two unknown carotenoids were only detected in group II and III.
